# Transfusion-transmitted malaria in sub-Saharan Africa: a systematic review and meta-analysis of *Plasmodium* spp. carriage in blood units and confirmed transmission in recipient

**DOI:** 10.1186/s12936-026-05975-0

**Published:** 2026-07-07

**Authors:** Lambert Morisho Mulakwa, Archippe Muhandule Birindwa, Chérone Nancy Mbani Mpega Ntigui, Justin Byamungu Ahadi, Patrick Ntagereka Bisimwa, Sandrine Lydie Oyegue Liabagui

**Affiliations:** 1 Regional Doctoral School of Central Africa in Tropical Infectious Diseases, Université des Sciences et Techniques de Masuku (USTM), BP 876, Franceville, Gabon; 2 Molecular Biology Laboratory, Université Évangélique en Afrique (UEA), BP 3323, Bukavu, Democratic Republic of Congo; 3 Faculty of Medicine, Université des Sciences et Techniques de Masuku (USTM), BP 914, Franceville, Gabon; 4 Department of Biology, Faculty of Science, Université des Sciences et Techniques de Masuku, (USTM), BP 914, Franceville, Gabon; 5 Evolution, Epidemiology and Parasitic Resistance Unit, Centre Interdisciplinaire de Recherches Médicales de Franceville (CIRMF), BP 769, Franceville, Gabon; 6 Preventive and Curative Services, Biomedical Research Unit, Vaccine and Antimicrobial Resistance, Centre de Santé Muhungu-État/CELPA, BP 1247, Bukavu, Democratic Republic of Congo

**Keywords:** Transfusion-transmitted malaria, *Plasmodium* spp. carriage, Blood donors, Sub-Saharan Africa, Systematic review

## Abstract

**Background:**

Transfusion-transmitted malaria (TTM) remains a major concern in malaria-endemic regions of Sub-Saharan Africa (SSA), where routine screening of blood donors for *Plasmodium* species is rarely implemented. Asymptomatic carriers among donors serve as silent reservoirs of infection, which puts transfusion recipients at substantial risk. This systematic review and meta-analysis aimed to assess the prevalence of *Plasmodium* spp. carriage among blood donors and evaluate the associated risk of TTM across SSA.

**Methods:**

A comprehensive literature search was conducted in PubMed (MEDLINE), Google Scholar, ScienceOpen, MedRxiv and BioRxiv for studies published up to July 2025. Eligible studies were those reporting the prevalence of *Plasmodium* spp. among blood donors or documenting cases of post-transfusion malaria in recipients. Data extraction was performed in accordance with PRISMA guidelines. The methodological quality of the included studies was independently assessed by two reviewers using the Joanna Briggs Institute (JBI) checklist and the AXIS tool for cross-sectional studies. A random-effects meta-analysis was performed using MetaAnalysis Online to estimate pooled prevalence, and publication bias was explored using Egger’s test.

**Results:**

A total of 1,364 records were identified, of which 12 studies met the inclusion criteria, representing 11,818 participants across West, Central, and East/Southern Africa. The pooled prevalence of asymptomatic *Plasmodium* spp. carriage among blood donors was 12% (95% CI 6–20%), based on a random-effects model, heterogeneity was substantial (I^2^ = 99%, p < 0.01), while Egger’s test (p = 0.581) indicated no significant publication bias. Three studies reported post-transfusion follow-up data. Comparative risk analysis revealed pronounced discrepancies between parasite prevalence in transfused blood and the number of documented TTM cases, with transmission rates among infected blood units ranging from 63% to 100%.

**Conclusion:**

Asymptomatic *Plasmodium* spp. carriage among blood donors poses a potential threat to transfusion safety in sub-Saharan Africa, underscoring the need for improved screening, strengthened haemovigilance, and further research.

**Supplementary Information:**

The online version contains supplementary material available at https://doi.org/10.1186/s12936-026-05975-0.

## Introduction

Blood transfusions are a vital medical intervention, particularly in setting where resource are limited. According to the World Health Organization (WHO), more than 118 million blood donations are collected globally each year [1, 2]. Nevertheless, Sub-Saharan Africa (SSA) continues to face frequent shortages in the availability of safe blood. While transfusions are essential for saving lives in cases of severe anaemia, obstetric haemorrhage or trauma, they also pose the risk of transmitting infectious agents, such as HIV, hepatitis B and C, syphilis and malaria parasites (*Plasmodium* spp.) [3–5]. Among these transfusion-transmitted infections, malaria is of particular concern in endemic regions where asymptomatic parasitaemia among blood donors is common and screening strategies are limited [5]. Malaria continues to be one of the leading causes of morbidity and mortality in SSA, where over 90% of the world's malaria cases and deaths occur. In 2022 alone, the WHO estimated 249 million malaria cases and 608,000 malaria-related deaths worldwide, more than 94% of which occurred in Africa. The majority of these deaths affected children under five years and pregnant women [6, 7].

While the overall burden of malaria underscores the magnitude of the challenge, its potential transmission through blood transfusions introduces an additional layer of complexity, particularly in fragile health systems. In such contexts, transfusion-transmitted malaria (TTM) presents both systemic challenges and medical risks [8]. These challenges are further intensified by weak infrastructure, conflict-related disruptions, and reliance on family replacement donors, all of which increase the likelihood of silent carriage of the *Plasmodium* parasite [9–13]. This highlights the urgent need to strengthen blood safety strategies in malaria-endemic countries.

Despite TTM being a recognised threat, the implementation of systematic screening remains inconsistent across SSA. Although affordable rapid diagnostic tests (RDTs) are available and awareness of transfusion-related risks has increased, malaria screening is not yet a routine practice in most blood banks or hospitals [14].Where screening does occur, limitations in test sensitivity and logistical barriers often prevent the consistent identification and removal of infected units [15, 16].In situations of chronic blood shortage, non-recommended practices may also emerge. For instance, blood donations that test positive for *Plasmodium* spp. are occasionally transfused in emergencies, particularly when the lives of children or mothers are at risk [14, 17].

Consequently, the true burden of TTM remains poorly quantified. Existing studies have reported the prevalence of *Plasmodium* spp. among blood donors to range from less than 2% in urban, lower-transmission areas, to over 25% in rural or peri-urban, endemic zones [10, 18–21]. However, only a few studies have systematically assessed residual risk by confirming post-transfusion parasitaemia in recipients [9, 22, 23]. This diagnostic and epidemiological gap perpetuates unmeasured transmission chains and exposes vulnerable populations to preventable infection.

Closing these gaps is essential to improve transfusion safety in malaria-endemic regions. This systematic review and meta-analysis summarises the available evidence on the prevalence of malaria parasites in donated blood and on confirmed cases of post-transfusion malaria in SSA. It also evaluates the quality and design of existing studies, highlighting key methodological shortcomings and providing evidence-based recommendations to support safer transfusion practices.

## Methods

### Study design and eligibility criteria

This systematic review and meta-analysis were conducted in accordance with the Preferred Reporting Items for Systematic Reviews and Meta-Analyses (PRISMA) 2020 guidelines [24]. The study protocol was registered in the PROSPERO database under the identifier CRD42025106123, ensuring methodological transparency [25]. The following study designs were eligible: cross-sectional studies, cohort studies, case series, case–control studies and surveillance reports, provided populations exposed to blood transfusion in SSA were included.

Originally, the protocol focused exclusively on studies conducted in the Democratic Republic of Congo (DRC). However, due to the limited number of studies available from this country, the scope was expanded to include the entire SSA region, in order to obtain a sufficient number of studies for a robust meta-analysis. This modification was carefully considered to ensure that the protocol remained both relevant and methodologically rigorous. Eligible populations included blood donors (whether voluntary, family or replacement), stored blood units intended for transfusion and patients who had received a transfusion. Studies were included if they reported the screening or detection of *Plasmodium* spp. in the blood prior to transfusion using validated diagnostic methods, such as microscopy, RDTs, and polymerase chain reaction (PCR) or equivalent tools. The analysis included publications in English or French that were published between January 2000 and May 2025. Conversely, articles that did not contain original data, such as literature reviews or editorials, were excluded. Studies that were not conducted on human subjects, those that were conducted outside SSA, or those that did not have a direct link to TTM were also excluded.

### Search strategy

A comprehensive literature search was conducted using the following databases: PubMed (MEDLINE), Google Scholar, ScienceOpen, MedRxiv, and BioRxiv. These data were complemented by grey literature sources, including reports from the WHO, national blood transfusion services, and non-governmental organizations (NGOs) operating in SSA. The aim was to enhance knowledge and understanding of the overall risk of infection associated with blood transfusions. The search strategy combined keywords and Medical Subject Headings (MeSH) terms such as ‘‘blood transfusion’’, ‘‘malaria’’, ‘‘*Plasmodium* spp.’’ and ‘‘Africa south of the Sahara’’, which were adapted for each database using Boolean operators (‘‘AND’’ and ‘‘OR’’). The full PubMed search strategy is provided in Supplementary Material 1.

To identify any additional eligible studies, the reference lists of the included articles and relevant reviews were also screened manually. The identified data were first examined through an initial review of the titles before being imported into the Zotero library. Duplicates were then removed to create a unified reference database, which was subsequently imported into the Rayyan software for the study selection phase [26, 27]. The literature search, selection and data extraction were conducted between November 2024 and May 2025. A critical reassessment of the review in July 2025 identified one additional eligible study (Noubouossie et al.) [13] which was included, to ensure that recent scientific advances was not overlooked. The final database was closed for analysis on 5th September 2025.

### Study selection, data handling and analytical stratification

**Fig. 1:**
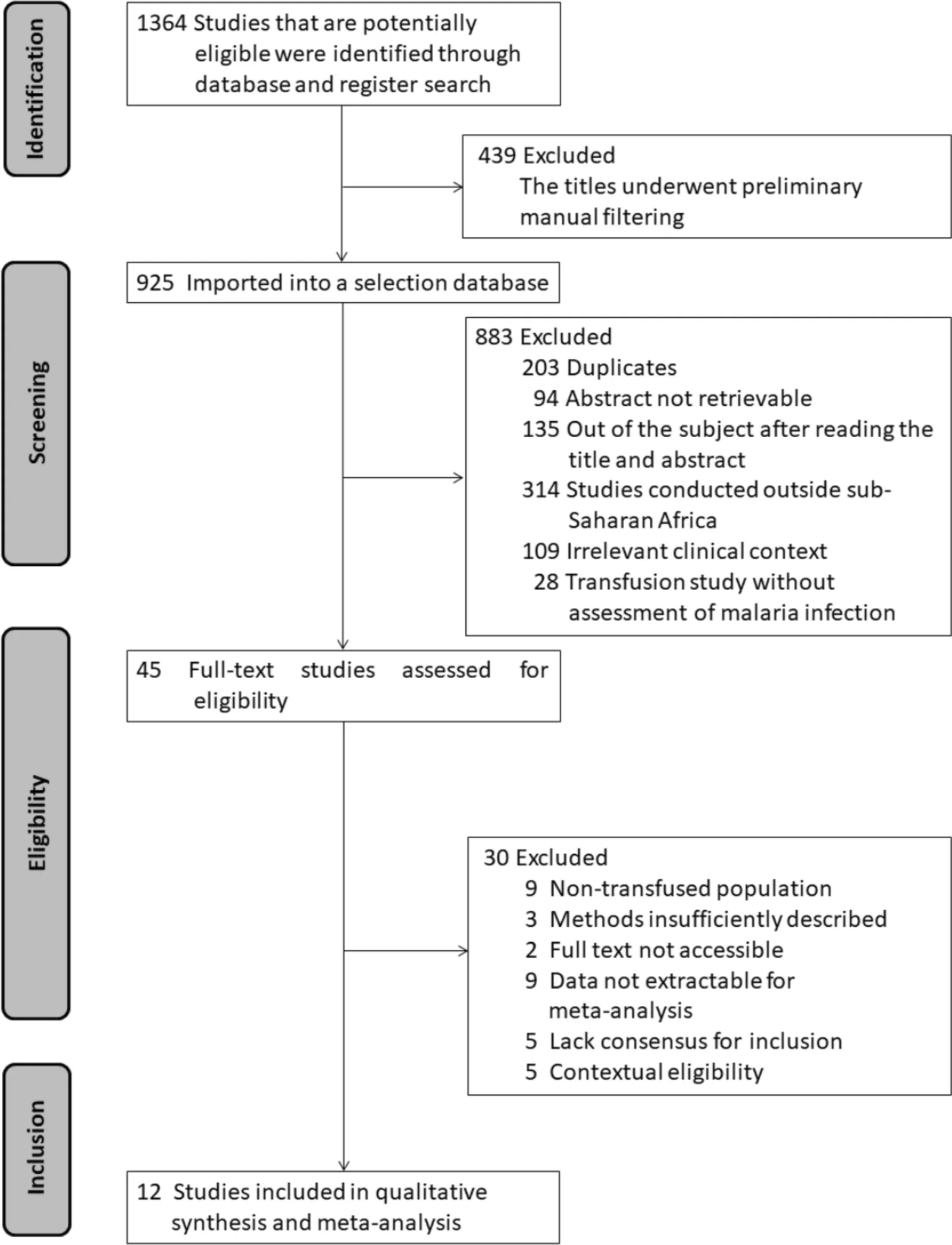
PRISMA flow diagram of study selection for the systematic review. Flowchart illustrating the identification, screening, eligibility assessment, and inclusion process of studies in this systematic review and meta-analysis

A progressive screening process based on predefined inclusion and exclusion criteria was then applied. The selected articles were independently reviewed by two assessors (LMM and JBA), who first screened the titles and abstracts followed by full-text evaluation of studies deemed potentially relevant before final inclusion. Any disagreements regarding study eligibility were resolved through consensus. This rigorous selection process is presented in the form of a PRISMA 2020 flowchart (Fig. 1). To avoid overestimating the prevalence or duplicating results, each study was carefully examined to identify the most sensitive diagnostic method.

Only one result per outcome was retained for analysis, following a predefined hierarchy of diagnostic accuracy: PCR was prioritized, followed by ELISA pan-LDH, Sysmex XN-31, microscopy, and finally RDTs [28, 29]. This approach ensured consistency and comparability across studies. Several studies have reported the detection of *Plasmodium* spp. in blood units prior to transfusion, as well as subsequent monitoring of recipients after transfusion. To maintain analytical coherence, two distinct datasets were constructed: one for the prevalence of *Plasmodium* spp. in the donated blood, and another for the number of confirmed cases of TTM. Where both datasets originated from the same study, the data were split accordingly with appropriate cross-referencing. Studies comparing blood donors and non-donors were included, but only the blood donor data was taken into account. Finally, all eligible studies were grouped into three epidemiological regions: West, Central and East/Southern Africa based on shared ecological and malaria transmission dynamics. This regional stratification enhances the interpretability of regional variations in transfusion-related malaria prevalence and supports the development of context-specific recommendations for malaria-endemic settings.

### Quality assessment and risk of bias appraisal

To ensure methodological rigour, a dual critical appraisal strategy was employed. This involved using the Joanna Briggs Institute (JBI) checklist, which was adapted for each study type, as well as the AXIS tool for cross-sectional studies. [30–32]. For non–peer-reviewed sources, including preprints and institutional reports, the same structured appraisal tools were applied without modification to ensure consistency of evaluation across publication types. The absence of peer review was explicitly considered as a potential source of methodological uncertainty during appraisal. Final scores from both tools were converted into percentages to facilitate inter-study comparison. These scores were used to classify the studies as having low, moderate or high methodological quality, defined as below 50%, 50–79% and 80% or above, respectively. In accordance with the study protocol, only studies achieving a quality score of at least 80% (i.e. 16/20 or 8/10, depending on the assessment tool) were included in the final synthesis. In addition to the numerical threshold, studies were required to present clearly described methodology, extractable primary outcome data, and internally coherent analytical procedures to be eligible for inclusion.

To minimise bias arising from incomplete reporting and to improve the accuracy of extracted data, corresponding authors were contacted when essential methodological details such as the precise data collection period were missing or unclear (Okalla Ebongue et al.) [33]. This proactive clarification step strengthened the reliability of the data and was applied in line with PRISMA 2020 recommendations. For preprints, additional verification focused on methodological transparency, completeness of reported outcomes, and consistency between objectives, methods, and results to mitigate risks associated with non–peer-reviewed dissemination. This mixed-methods approach enhanced the objectivity of the risk of bias assessment and reduced evaluator subjectivity. The consistent application of this procedure prevented over-representation and ensured robust data extraction. The combined use of JBI and AXIS tools enabled for a comprehensive evaluation of the internal validity, statistical reliability, and overall transparency of the included studies.

### Data analysis and meta-analysis

The data were initially compiled in Microsoft Excel 2016 and subsequently exported for quantitative synthesis using MetaAnalysisOnline (© metaanalysisonline.com, 2024–2025) [34]. This tool allows for the standardised entry of study-level data including sample size, number of events and effect sizes. It also calculates pooled estimates using random-effects models given the substantial heterogeneity across studies, particularly with regard to design, diagnostic methods and sample size, a random-effects model (DerSimonian–Laird method) was selected to generate more reliable and generalisable results. Key outputs included a forest plot summarising the pooled prevalence of *Plasmodium* spp. carriage among blood donors or units, a funnel plot to assess potential publication bias and heterogeneity statistics, such as Cochran's Q and the I^2^ index [34]. To complement this meta-analysis and further explore the descriptive patterns of transfusion-transmitted malaria (TTM), a comparative analysis of the relationships between Plasmodium-positive blood units, transfused recipients, and confirmed TTM cases was conducted.

This analysis explored the relationships among three keys variables: the number of *Plasmodium* spp. positive blood units, the number of transfused patients and the number of confirmed TTM cases. A scatterplot matrix was generated to visualise all variable pairs and identify potential associations. The statistical analysis was performed using DATAtab (DATAtab Team, 2025), a free web-based statistical tool. The analysis was based on three published studies conducted in Benin, Nigeria and Mali (Anani et *al.*, Iheonu et *al.* and Loua et *al.*) [9, 22, 23]. The following quantitative variables were extracted from these studies: the number of blood units that were tested positive for *Plasmodium* spp.; the number of patients who received transfusions and the number of confirmed cases of post-transfusion malaria. This focused dataset enabled a targeted examination of the risk of TTM in endemic contexts.

## Results

The PRISMA diagram summarises how studies were selected for inclusion in this systematic review and meta-analysis (Fig. 1). A total of 1364 records were initially identified through database searches and other sources. Following an initial reading of the titles, 439 were excluded. A further 883 were excluded after a more detailed assessment for various reasons (duplicates, missing abstracts, or irrelevance). The full texts of 42 articles were retained for eligibility assessment. Of these, 30 were excluded for various reasons (Supplementary Materiel 2 and 3). Ultimately, 12 studies met all the inclusion criteria and were selected for qualitative and quantitative synthesis.

### The geographic distribution of the included studies across SSA

**Fig. 2:**
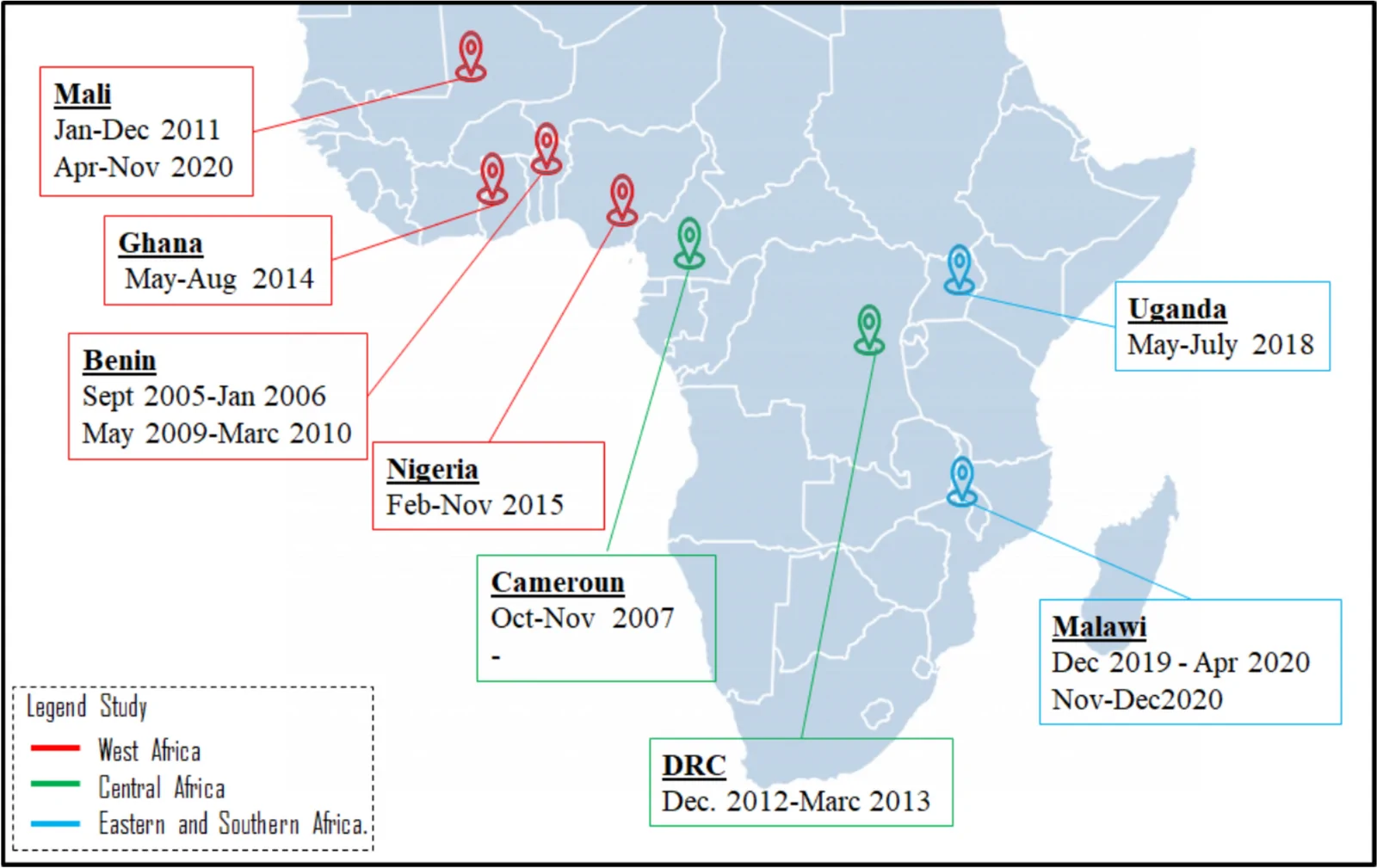
Geographic mapping of included studies of *Plasmodium* spp. carriage in blood donors and TTM in SSA. Map illustrating the spatial distribution of studies included in the systematic review and meta-analysis, highlighting countries where donor parasitaemia and/or confirmed TTM cases were reported

The geographical locations and time periods covered by the twelve studies included in this systematic review are represented on a map (Fig. 2). The countries of West Africa (Nigeria, Ghana, Mali and Benin) [9, 17, 18, 22, 23, 35] are the most represented, while those of Central Africa (Cameroon and DRC) [10, 13, 33] and East/South Africa (Uganda and Malawi) are underrepresented [19, 20]. The field studies span 15 years, with the earliest study dating back to 2005 and the most recent one conducted in 2020. While this overview shows all the research conducted across the SSA region, there are some notable gaps. Despite malaria being highly prevalent in many parts of Africa, researchers have produced limited results, highlighting disparities in the structural and logistical aspects of malaria surveillance systems. The twelve studies each collected data during their respective collection periods, and were grouped into three categories based on regional climatology and transmission patterns. The studies conducted during periods of high transmission (n = 6): Kumasi, Akatsi, Ouémé-Atlantique-Littoral, Lagos, Bamako (2020), Kampala-Jinja; those conducted during moderate or transitory periods (n = 4): Kisangani, Bamako (2011), All States Nigeria and Cotonou; and those covering areas of stable or perennial transmission (n = 2): Douala and Yaoundé.

### Timeline of scientific publications on *Plasmodium* spp. research in blood transfusion

**Fig. 3:**
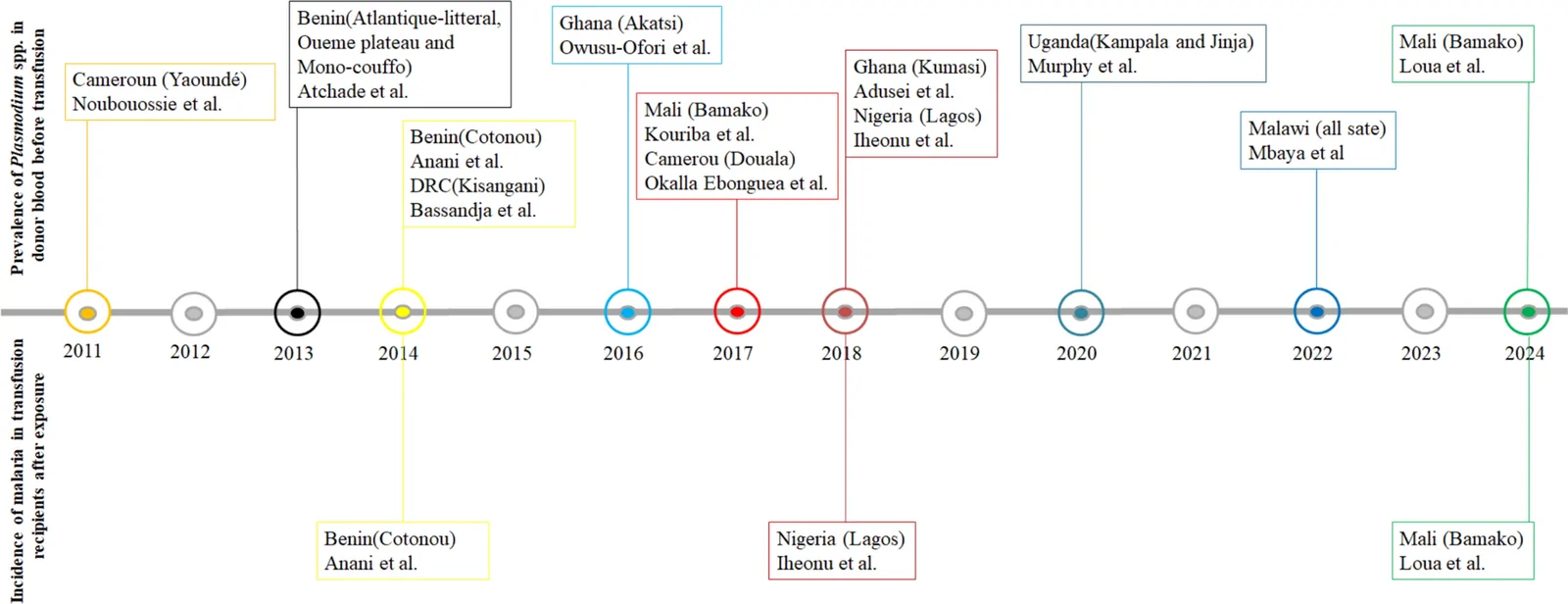
Timeline of scientific publications on *Plasmodium* spp. research in blood transfusion in SSA. Graph illustrating the temporal distribution of studies included in the review, showing the number of publications per year. Timeline showing publication trends and periods of limited research activity in transfusion-related malaria studies

The chronological overview of the years in which the 12 included studies were published is provided. The studies are categorised as focusing either on *Plasmodium* spp. carriage in blood units (above the line) or on documented transmission in transfusion recipients (below the line). The country, city, province or locality in which the data for each study were collected is specified (Fig. 3). From 2000 to 2025, publication rates were irregular, alternating between periods of inactivity and periods of more intense publication. For instance, a period of inactivity occurred between 2000 and 2010, followed by periods of increased publication activity between 2013 and 2018, and again between 2020 and 2024. However, there were also years with no publications, including 2012, 2015, 2019, 2021 and 2023. Of the 12 studies, which all focused on donor screening, only three reported confirmed cases of TTM in recipients, highlighting a significant imbalance. This underscores the scarcity of systematic post-transfusion follow-up and the absence of routine surveillance mechanisms to confirm TTM, despite malaria's high endemicity.

### Study characteristics and methodological quality

A total of 12 studies on *Plasmodium* spp. carriage in blood donors and TTM were identified across sub-Saharan Africa. These studies involved a total of 11,818 participants (see Table 1). Sample sizes ranged from 100 to over 5000 participants, and most studies were conducted in hospitals (n = 7) or blood transfusion centres (n = 5). The designs included cross-sectional surveys, prospective cohorts and diagnostic evaluations, with microscopy being the predominant diagnostic tool, followed by ELISA, PCR, RDTs and automated platforms. Methodological quality was generally acceptable, with reported appraisal scores ranging from 80 to 100% when assessed using the AXIS and/or JBI tools.

**Table 1:** Summary of study characteristics, diagnostic approaches, and appraisal scores for included articles

Authors and publication year	Region	Country	Study duration (months)	Study setting	Study population	Sample size	Diagnostic tool	Study design	Appraisal strategy	Score (%)
Adusei et al (2018) [17]	West Africa	Ghana	8	Hospital	Donor	100	Microscopy	Cross-sectional descriptive	AXIS	87.5
Anani et al (2014) [22]	West Africa	Benin	4	Hospital	Donor–Recipient	141	Microscopy	Prospective observational	JBI–Cohort	80
Atchade et al (2013) [35]	West Africa	Benin	10	Blood transfusion center	Donor	2515	ELISA	Cross-sectional diagnostic evaluation	JBI–Diagnostic Accuracy	100
Bassandja et al (2014) [10]	Central Africa	DRC	4	Blood transfusion center	Donor	480	Microscopy	Cross-sectional descriptive	AXIS	87.5
Iheonu et al (2018) [23]	West Africa	Nigeria	9	Hospital	Donor–Recipient	164	Microscopy	Prospective with follow-up	JBI–Cohort	82.5
Kouriba et al (2017) [18]	West Africa	Mali	11	Blood transfusion center	Donor	946	Microscopy	Cross-sectional descriptive	AXIS	80
Loua et al (2024) [9]	West Africa	Mali	7	Hospital	Donor–Recipient	348	Microscopy	Analytical observational cross-sectional	JBI–Analytical X-sec	90
M'baya et al (2022) [19]	East & Southern Africa	Malawi	7	Blood transfusion center	Donor	5281	Sysmex (automated)	Prospective diagnostic evaluation	JBI–Diagnostic Accuracy	100
Murphy et al (2020) [20]	East & Southern Africa	Uganda	2	Blood transfusion center	Donor	1000	PCR	Cross-sectional + multivariate analysis	JBI–Analytical X-sec	85
Noubouossie et al. (2011) [13]	Central Africa	Cameroon	2	Hospital	Donor	493	Microscopy	Cross-sectional	AXIS	90
Okalla Ebongue et al. (2017) [33]	Central Africa	Cameroon	–	Hospital	Donor	250	Microscopy	Descriptive + risk modelling	JBI–Analytical X-sec	87.5
Owusu-Ofori et al. (2016) [36]	West Africa	Ghana	3	Hospital	Donor	100	RDTs	Cross-sectional	AXIS	85

AXIS: Appraisal tool for Cross-Sectional Studies; JBI: Joanna Briggs Institute checklist*;* PCR: Polymerase Chain Reaction; ELISA: Enzyme-Linked Immunosorbent Assay; RDTs: Rapid Diagnostic Tests; Sysmex (automated): Automated hematology of *Plasmodium* spp. diagnostic; DRC: Democratic Republic of the Congo

### Pooled prevalence of *Plasmodium* spp. carriage among blood donors in sub-Saharan Africa

**Fig. 4:**
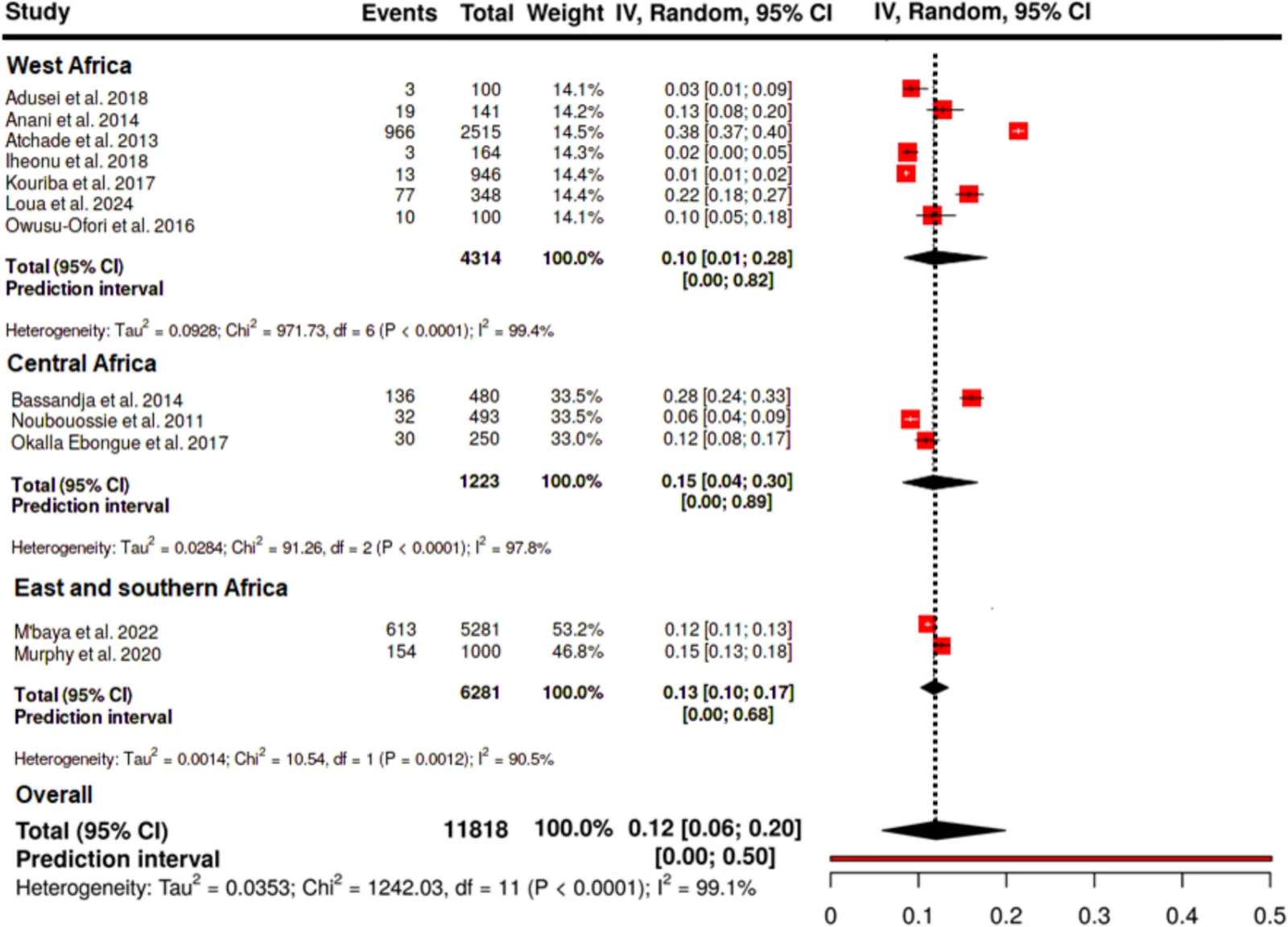
Pooled prevalence of *Plasmodium* spp. carriage in blood donors: Forest plot from the meta-analysis (n = 12 studies). Events refer to the number of individuals with the outcome of interest (e.g., malaria infection) in each study. Weight represents the statistical contribution of each study to the pooled estimate. IV, Random indicates the inverse-variance method under a random-effects model. CI denotes confidence interval. Tau^2^ (Tau-squared) estimates between-study variance under the random-effects model. Chi^2^ (Chi-squared) tests for statistical heterogeneity across studies. df indicates degrees of freedom. I^2^ quantifies the proportion of total variation attributable to heterogeneity rather than chance. A p-value < 0.05 is considered statistically significant. Forest plot displaying individual study prevalence estimates and the overall pooled estimate calculated using the inverse-variance random-effects model. Each study is represented by a red square proportional to its statistical weight, with horizontal lines indicating the 95% confidence interval (CI). The black diamond at the bottom represents the overall pooled prevalence with its corresponding 95% CI

**Fig. 5:**
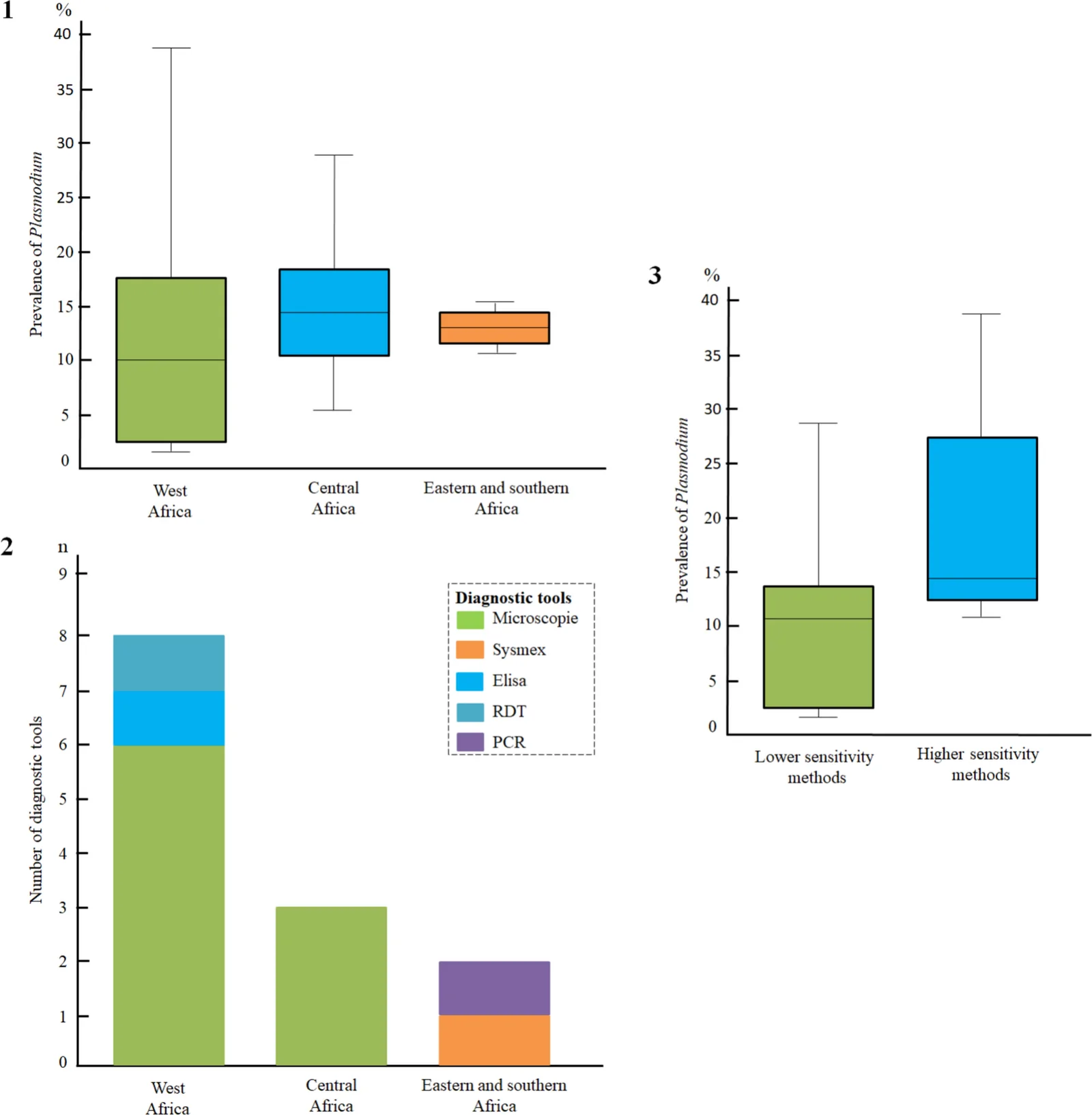
Distribution of malaria prevalence and diagnostic methods across sub-Saharan Africa. Lower-sensitivity methods include microscopy and rapid diagnostic tests. Higher-sensitivity methods include PCR, ELISA, and Sysmex-based detection. Prevalence values represent those reported in the included studies. RDT = Rapid Diagnostic Test; PCR = Polymerase Chain Reaction; ELISA = Enzyme-Linked Immunosorbent Assay. (1) Boxplot showing the distribution of reported malaria prevalence across West, Central, and Eastern/Southern Africa. (2) Distribution of diagnostic tools used across regions, illustrating the predominance of microscopy compared to Sysmex, ELISA, RDT, and PCR. (3) Comparison of reported prevalence according to diagnostic sensitivity, grouped into lower-sensitivity methods (microscopy and RDT) and higher-sensitivity methods (PCR, ELISA, Sysmex)

In total, 12 studies were included in the meta-analysis, with a cumulative sample size of 11,818 participants (Fig. 4). The analysis was conducted using a random-effects model with the inverse variance method, combined with the Freeman–Tukey double arcsine transformation to stabilize the variance of observed proportions. The pooled prevalence of *Plasmodium* spp. carriage among blood donors was estimated at 0.12 (12%), with a 95% confidence interval ranging from 0.06 to 0.20 (Fig. 5). A statistically significant heterogeneity was observed (p < 0.01), indicating substantial variability across the included studies. The I^2^ statistic was 99%, suggesting that most of the observed variability reflects between-study differences rather than random error. Given this level of heterogeneity, the pooled estimate represents a summary measure across heterogeneous study contexts. Study-level prevalence estimates varied widely, reflecting differences in diagnostic methods, study settings, and epidemiological conditions.

### Distribution of diagnostic methods and corresponding malaria prevalence across African regions

In Fig. 5, microscopy was the most frequently used diagnostic method, reported in 8 of the 12 included studies (66.7%), with most studies conducted in West Africa (5 studies), followed by Central Africa (3 studies). In contrast, ELISA, PCR, RDTs, and Sysmex-based methods were each used in only one study, representing 8.3% of studies per method. Overall, non-microscopy diagnostic approaches accounted for 4 of the 12 studies (33.3%). Across all diagnostic methods, the mean prevalences reported ranged from approximately 2–18%. Microscopy showed the widest range of prevalence estimates, from 1.4% to 28.3%, ELISA, PCR, RDTs, and Sysmex their respective prevalence is 38.4%, 15.4%, 10%, and 11.6%. When grouped according to analytical sensitivity, studies using microscopy or RDTs (lower sensitivity methods) reported a mean prevalence of 10.9%, whereas studies using PCR, ELISA, or Sysmex (higher sensitivity methods) reported a mean prevalence of 21.8%. However, this comparison remains descriptive given the limited number of studies in the higher-sensitivity group.

### Assessment of publication bias

**Fig. 6:**
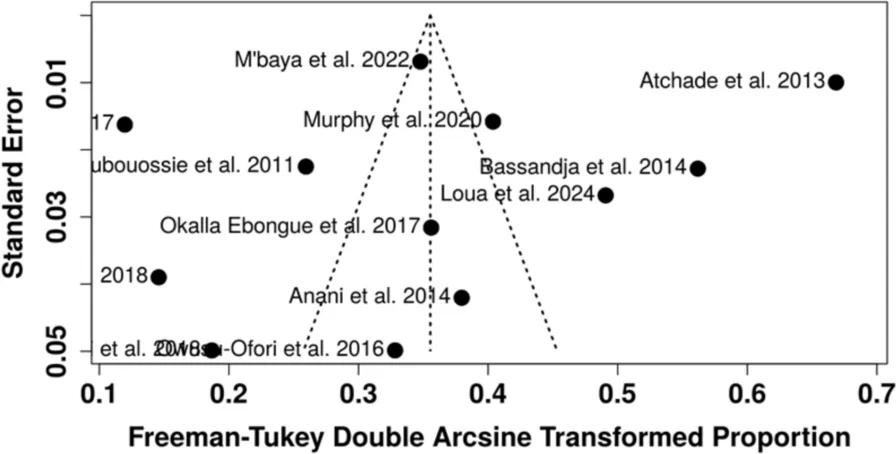
Funnel plot for publication bias assessment among the included studies. The funnel plot displays individual study effect estimates against their corresponding standard errors to evaluate potential publication bias or small-study effects in the meta-analysis. The symmetry of the funnel plot was visually inspected to assess potential small-study effects or publication bias. Asymmetry, particularly the relative absence of studies in specific regions of the plot, may suggest the presence of publication bias, substantial heterogeneity, or other small-study effects. However, given the very high between-study heterogeneity (I^2^ ≈ 99%), visual interpretation of funnel plot asymmetry should be undertaken with caution

To assess the presence of potential publication bias among the included studies, a funnel plot was generated (Fig. 6**)**. The visual inspection of the plot does not suggest any clear asymmetry, indicating a low likelihood of publication bias. This observation is further supported by Egger’s regression test, which did not show statistically significant evidence of small-study effects (intercept = −3.15; 95% CI −13.98 to 7.68; t = −0.571; p = 0.581). These findings suggest that the results of the meta-analysis are unlikely to be substantially influenced by selective publication or reporting.

### Comparative assessment of TTM risk in SSA

A descriptive comparison of three studies conducted in West Africa is presented in Table 2 (Anani et al., Loua et al., and Iheonu et al.) [9, 22, 23]. All studies screened donated blood for *Plasmodium* spp. using microscopy and followed transfusion recipients for post-transfusion malaria. In all three studies, parasitised blood units were identified. Marked discrepancies were observed between the prevalence of *Plasmodium*-positive blood bags and the number of confirmed post-transfusion malaria cases. Contamination rates of donated blood exceeded 50% in all studies, with one study conducted in a neonatal setting in Nigeria reporting a transmission rate greater than 100% (133%). The number of confirmed TTM cases did not consistently mirror the prevalence of infected blood bags across studies, highlighting substantial variability in reported outcomes [23].

**Table 2:** Comparative analysis of TTM risk in three SSA studies

Authors and publication year	Sample size	Prevalence of positive blood bags (%)	Positive blood bags (Cases)	Transfused cases	Confirmed TTM cases	TTM prevalence (%)	Transmission rate among positive bags (%)	Risk analysis–Interpretation
Anani et al. (2014) [22]	141	13,47%	19	77	12	15,78%	63,2%	Probable TTM from identified infected bags → No prophylaxis; endemic context
Iheonu et al. (2018) [23]	164	1,8%	3	164	4	2,4%	133% ^*^	Surprising result: positive cases not strictly correlated with positive blood units → Undervalued risk
Loua et al. (2024) [9]	348	22%	77	20 ^**^	20	100%	100%	All recipients of the positive blood units developed malaria → Confirmed high risk

^*^A rate of 133% in Iheonu study may suggest the presence of biases, such as unidentified clusters of infections, uncontrolled external factors (e.g. errors in surveillance) or limitations in the detection tools used

^**^Follow-up of the 20 recipients transfused with *Plasmodium* spp.-infected blood bags was strictly carried out for Loua et al.; the other bags, whether contaminated or not, were not monitored

### Descriptive comparison of infected blood units and reported TTM cases in three SSA studies

**Fig. 7:**
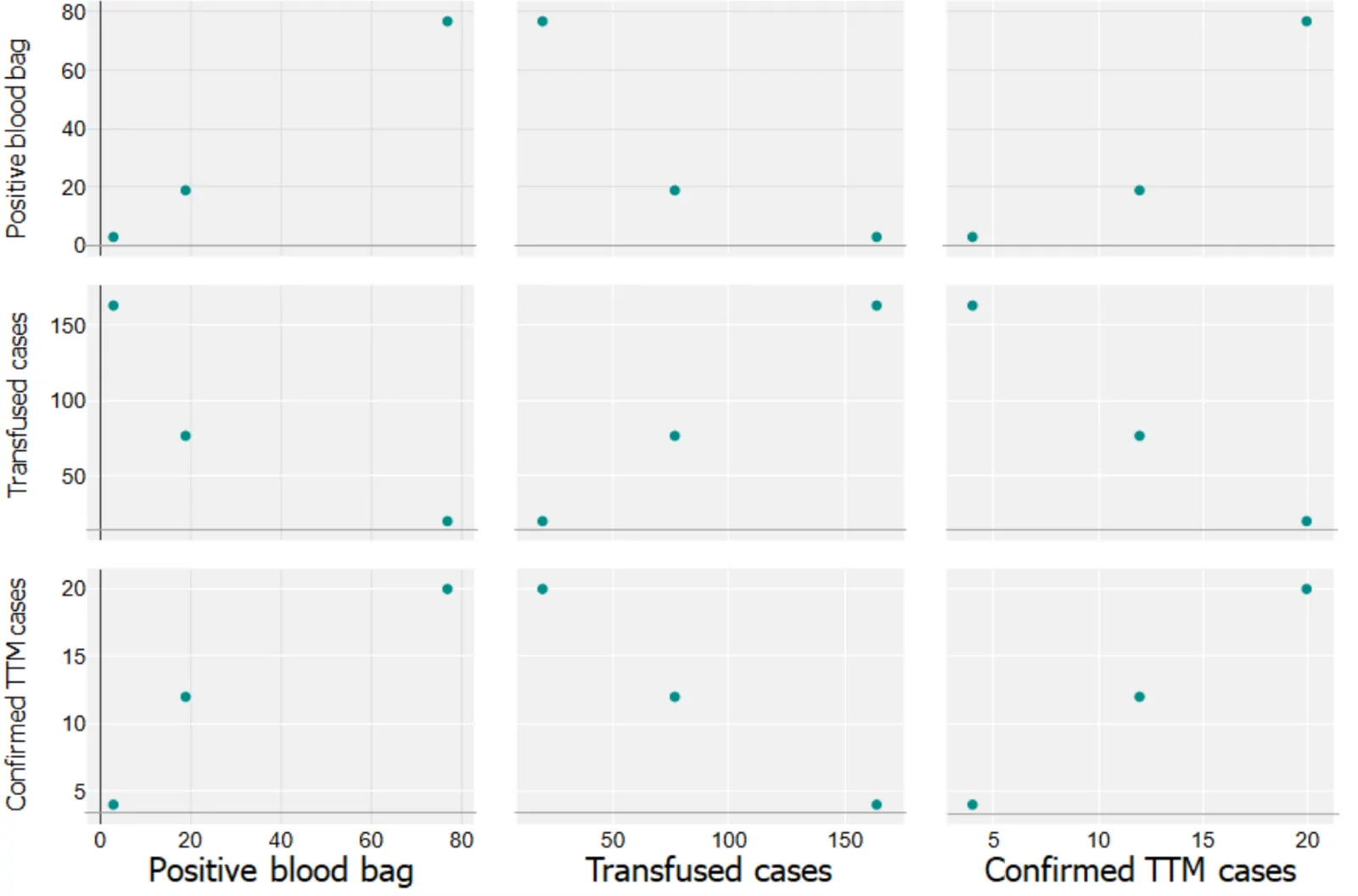
Descriptive comparison of infected blood units, transfused recipients, and confirmed TTM cases across three SSA studies. This figure presents a descriptive visual comparison of the number of *Plasmodium*-infected blood units, transfused recipients, and confirmed TTM cases reported in three studies conducted in sub-Saharan Africa. Each point represents one study. No statistical inference is intended

In order to explore the relationships between the number of *Plasmodium* spp.-positive blood units, the number of transfused recipients, and the number of confirmed cases of transfusion-transmitted malaria (TTM), a scatterplot matrix was generated (Fig. 7). This visual representation allows for a descriptive comparison of how these variables co-vary across the three available studies. Across the three studies, increases in the number of *Plasmodium*-positive blood units were accompanied by higher numbers of transfused recipients and confirmed TTM cases. However, the magnitude of these outcomes varied substantially between studies, indicating heterogeneity in reported transmission patterns. This analysis is presented for descriptive purposes only and aims to illustrate observed patterns within the scarce body of studies reporting both infected blood units and confirmed TTM cases, rather than to establish statistical associations or causal relationships.

## Discussion

This systematic review and meta-analysis sought to estimate the prevalence of *Plasmodium* spp. carriage among blood donors or units in sub-Saharan Africa (SSA), with an initial focus on the Democratic Republic of Congo (DRC), and to assess the available evidence regarding transfusion-transmitted malaria (TTM). Although 1,364 records were initially retrieved, only 12 studies met the inclusion criteria and were retained for qualitative and quantitative synthesis. This substantial reduction reflects both the scarcity of structured haemovigilance systems and the limited availability of recipient follow-up data in endemic settings. The pooled prevalence of *Plasmodium* spp. carriage among 11,818 blood donors was estimated at 12% (95% CI 6–20%), with very high heterogeneity (I^2^ = 99%). Given this extreme heterogeneity, the pooled estimate should be interpreted as a global descriptive indicator rather than a precise epidemiological parameter applicable uniformly across SSA settings. Such heterogeneity suggests considerable variability in methodological approaches, ecological contexts, seasonal timing, and diagnostic sensitivity rather than a single uniform epidemiological pattern across SSA. Only three studies reported data on transfusion recipients and confirmed cases of TTM, underscoring the absence of systematic post-transfusion surveillance. Descriptive cross-comparisons indicated concordant trends between malaria-positive blood units and reported TTM cases; however, no formal correlational or causal inference was performed. The limited number of recipient-based investigations, combined with the absence of molecular confirmation in most settings, indicates that TTM likely remains underdiagnosed in routine clinical practice [4], and therefore the true burden may be substantially underestimated.

Several methodological limitations must be acknowledged when interpreting these findings. First, there was a strong predominance of microscopy across included studies, while ELISA, PCR, rapid diagnostic tests (RDTs), and automated analysers such as Sysmex were each used in only one study. Microscopy, although widely accessible and affordable in endemic settings, has limited sensitivity for detecting low-density or subpatent infections [37, 38]. In contrast, molecular techniques such as PCR can identify infections missed by conventional microscopy [39, 40]. The sporadic use of higher-sensitivity diagnostics suggests that submicroscopic parasitaemia may be underestimated in routine donor screening. However, while molecular techniques such as PCR substantially improve sensitivity compared with microscopy, they also present inherent interpretative limitations. In particular, PCR may detect residual *Plasmodium* DNA from non-viable parasites in stored or processed blood samples, which could potentially lead to overestimation of active parasitaemia prevalence. Unlike microscopy, which confirms the presence of viable intraerythrocytic parasites, PCR-based assays amplify genetic material regardless of parasite viability. This dual characteristic complicates direct comparison between diagnostic modalities and adds further complexity to the interpretation of pooled prevalence estimates [41–43]. Reported mean prevalence varied across diagnostic methods, with broader ranges observed in microscopy-based studies compared with other techniques. However, given the small number of studies employing molecular or antigen-based assays, robust comparative inference is not possible. Differences in reported prevalence may reflect operator expertise, parasite density thresholds, and study timing rather than intrinsic diagnostic superiority alone, which calls for caution when interpreting apparent methodological differences. Second, the very high heterogeneity (I^2^ = 99%) indicates substantial between-study variability. Although a random-effects model was applied to account for this diversity, residual heterogeneity reduces the precision of pooled estimates. Beyond methodological variation, environmental and epidemiological factors contribute significantly to this variability. Seasonal transmission patterns strongly influence *Plasmodium* prevalence. Studies conducted during or shortly after rainy seasons in areas such as Kumasi, Akatsi, Bamako, Ouémé-Atlantique, Kampala-Jinja, and Lagos [9, 17, 20, 23, 35, 36] coincided with peak mosquito density and increased parasitaemia risk. In contrast, studies performed in Kisangani, Malawi, Cotonou, and Yaoundé [10, 13, 19, 22] took place during transitional or moderate transmission periods. Persistent year-round transmission in equatorial zones with stable breeding sites further complicates prevalence estimation [44, 45]. In regions such as Douala and parts of southern Cameroon, equatorial rainfall and high humidity sustain continuous transmission [46–49]. Failure to systematically adjust for seasonality likely amplifies inter-study heterogeneity, thereby limiting the comparability of prevalence estimates across settings.

Geographic distribution of studies was also uneven. Nearly two-thirds originated from West Africa, reflecting disparities in research infrastructure and funding. Conflict-affected and Francophone regions, including eastern DRC, Burkina Faso, Niger, and the Central African Republic, remain underrepresented despite high transmission intensity [50, 51]. Similarly, data from northern Angola and parts of Ethiopia remain scarce [50, 52–54]. The Malaria Atlas Project (MAP) identifies extensive zones of stable or intense transmission where robust transfusion-related data are lacking [55, 56]. This imbalance may limit generalisability and suggests potential publication and reporting bias, as areas with weaker research capacity may be systematically underrepresented in the literature. Grey literature, institutional haemovigilance reports, and unpublished hospital data were difficult to access, raising the possibility that negative or null findings remain underreported.

The design of the available and included studies also appears to contribute to the uncertainty. Most studies were cross-sectional and descriptive, focusing exclusively on donors. Few included systematic follow-up of recipients. Replacement donors, often recruited on an emergency basis, may undergo less rigorous screening and rely on self-reported medical histories, introducing memory and reporting bias [57–59]. In endemic areas, asymptomatic carriage is common [60–70] and screening strategies based solely on clinical history and temperature measurement are insufficient [15, 71]. Therefore, donor seroprevalence is often used as an indicator of transfusion risk, although donor infection does not automatically translate into clinical transmission. Parasite density, recipient immunity, transfusion volume, and environmental exposure all influence transmission dynamics [4, 48, 72–78], highlighting the multifactorial nature of transfusion-related risk.

Regional prevalence patterns reflect ecological variability. Central Africa exhibited the highest pooled estimate (15%), likely related to hot, humid equatorial climates and sustained transmission [60, 79, 80]. This elevated prevalence may be related to sustained high transmission intensity and greater background parasitaemia in the population. Differences in screening strategies, diagnostic sensitivity, and donor selection procedures may also partially explain this regional disparity. West Africa showed an average of 10%, influenced by seasonal rainfall patterns, while Southern/East Africa presented an intermediate estimate of 13%, consistent with its heterogeneous ecological context [81]. These differences likely reflect environmental and epidemiological diversity rather than intrinsic differences in transfusion practices alone, and should therefore not be interpreted as direct indicators of transfusion system performance.

The descriptive comparison between infected blood units and confirmed TTM cases should be interpreted cautiously. Post-transfusion malaria episodes may reflect nosocomial or community-acquired infection in highly endemic settings, particularly during prolonged hospitalisation [8, 9]. Surveillance artefacts and differences in follow-up intensity may lead to under- or overestimation of confirmed TTM cases. Misclassification bias is plausible in the absence of baseline parasitological assessment prior to transfusion. Moreover, biological dynamics complicate temporal attribution. Parasites may be present in donated blood at densities below detection thresholds or in developmental stages escaping routine screening. Following transfusion, parasites may proliferate in recipients transitioning from hepatic to erythrocytic phases, making precise attribution challenging [82]. These factors indicate that discrepancies between contaminated blood units and confirmed TTM cases likely reflect a complex interplay of epidemiological, methodological, and biological processes rather than a simple linear transmission relationship. Although descriptive concordance suggests a probable transmission link, donor prevalence alone is insufficient for precise risk quantification [71, 75, 83], and these findings should inform hypothesis generating recommendations rather than definitive policy guidance. Effective prevention requires context-adapted screening strategies, strengthened haemovigilance systems, expanded access to molecular diagnostics, and targeted prophylaxis for high-risk recipients, particularly in rural settings with limited laboratory infrastructure and electricity [4, 14, 84, 85]. These strategies need to be evaluated further to assess their feasibility, cost-effectiveness, and operational constraints.

Although this review was initially focused on the DRC, the scope was expanded to SSA due to limited national data. This broader approach enhanced statistical robustness and allowed regional stratification across comparable epidemiological contexts [22, 55, 86]. Despite differences in transfusion practices and malaria epidemiology between regions, stratified analysis improves interpretability. However, the limited number of recipient-based studies, methodological diversity, and scarcity of high-quality longitudinal data remain important constraints, and these factors should be considered when interpreting the pooled estimates.

Overall, the pooled prevalence of approximately 12% highlights transfusion-transmitted malaria as a persistent and under-recognised public health concern in endemic regions. Despite substantial heterogeneity and surveillance gaps, the evidence consistently indicates that asymptomatic donor carriage represents a non-negligible transfusion risk even if the exact magnitude of transmission cannot be precisely quantified. Future research should prioritise longitudinal recipient follow-up, molecular confirmation of transmission pathways, integration of sensitive diagnostic tools into routine screening, and the development of context-specific haemovigilance frameworks capable of accurately quantifying TTM burden in high-transmission settings. This can serve as a basis for testing potential interventions, rather than for the immediate implementation of policies.

## Conclusion

This systematic review and meta-analysis estimated a pooled prevalence of 12% for asymptomatic *Plasmodium* spp. carriage among blood donors in sub-Saharan Africa, highlighting a non-negligible potential risk of transfusion-transmitted malaria (TTM). Despite variability in study design, diagnostic approaches, and epidemiological contexts, contaminated blood units were consistently reported across regions, suggesting that donor parasitaemia remains a persistent concern in endemic settings.

However, these findings must be interpreted cautiously. The evidence base is limited by high heterogeneity (I^2^ = 99%), predominance of cross-sectional donor-focused studies, scarce recipient follow-up data, and uneven geographic representation. Differences in diagnostic sensitivity and seasonal transmission patterns further restrict comparability and may influence prevalence estimates. Consequently, precise quantification of transfusion-attributable risk remains challenging.

Although definitive policy recommendations cannot be drawn, the results underscore the need for strengthened haemovigilance systems, improved diagnostic strategies, and well-designed longitudinal studies including recipient monitoring. These findings may inform future research and support context-adapted strategies aimed at enhancing transfusion safety for vulnerable populations in malaria-endemic regions.

## Supplementary Information

Supplementary Material 1.

Supplementary Material 2.

Supplementary Material 3.

## Data Availability

All data extracted and analyzed in this review are contained within the manuscript or supplementary files. Additional materials can be provided upon request.
