# Supplementary material for: Transfusion-transmitted malaria in sub-Saharan Africa: a systematic review and meta-analysis of Plasmodium spp. carriage in blood units and confirmed transmission in recipient
Source: Malar J. 2026 Jul 7;25:343. doi: 10.1186/s12936-026-05975-0 (PMC13625347; doi:10.1186/s12936-026-05975-0)
Supplement: Supplementary file 1 [file 12936_2026_5975_MOESM1_ESM.docx]

**Supplementary Material 1**

**Full PubMed Search Strategy**

**Database:** PubMed (MEDLINE)
**Date of search:** 2000-2025
**Filters applied:** Humans; English and French

**Complete Boolean Search String**

(("Blood Transfusion"[Mesh] OR "blood transfusion" OR "transfusion-transmitted" OR "transfusion-transmitted malaria" OR "TTM" OR "blood donor*" OR "donated blood" OR "blood unit*")) AND (("Malaria"[Mesh] OR malaria OR "Plasmodium"[Mesh] OR "Plasmodium spp." OR "Plasmodium falciparum" OR "Plasmodium vivax" OR "Plasmodium malariae" OR "Plasmodium ovale")) AND (("Africa South of the Sahara"[Mesh] OR "Sub-Saharan Africa" OR "SSA" OR "West Africa" OR "East Africa" OR "Central Africa" OR "Southern Africa" OR Angola OR Benin OR Botswana OR Burkina Faso OR Burundi OR Cameroon OR Cape Verde OR Central African Republic OR Chad OR Comoros OR Congo OR "Democratic Republic of Congo" OR Djibouti OR Eritrea OR Eswatini OR Ethiopia OR Gabon OR Gambia OR Ghana OR Guinea OR "Guinea-Bissau" OR Kenya OR Lesotho OR Liberia OR Madagascar OR Malawi OR Mali OR Mauritania OR Mauritius OR Mozambique OR Namibia OR Niger OR Nigeria OR Rwanda OR Senegal OR Seychelles OR Sierra Leone OR Somalia OR "South Africa" OR South Sudan OR Sudan OR Tanzania OR Togo OR Uganda OR Zambia OR Zimbabwe))
