# Supplementary material for: Transfusion-transmitted malaria in sub-Saharan Africa: a systematic review and meta-analysis of Plasmodium spp. carriage in blood units and confirmed transmission in recipient
Source: Malar J. 2026 Jul 7;25:343. doi: 10.1186/s12936-026-05975-0 (PMC13625347; doi:10.1186/s12936-026-05975-0)
Supplement: Supplementary file 2 [file 12936_2026_5975_MOESM2_ESM.docx]

**Supplementary Material 2**

**Explanatory Note on the Extraction of Selected Articles (Mutually Exclusive Version)**

All articles selected for full-text evaluation underwent a structured data extraction process conducted independently by members of the evaluation team. Conflicts were resolved by consensus between two reviewers, with a third reviewer involved if necessary. Studies were evaluated against predefined inclusion criteria, including population, diagnostic methods, and transfusion-related outcomes.

Articles not meeting the requirements for quantitative synthesis were classified into seven **mutually exclusive exclusion categories**, based on methodological, clinical, or scientific considerations. This ensured transparency, consistency, and reproducibility prior to the final meta-analysis.

**Exclusion Categories :**

1. **No transfused population / irrelevant**

**Description:** Studies that do not include human transfusion recipients or assess transfusion outcomes. Examples: studies on costs, logistics, training, knowledge or practices of blood donation, behavioral assessments, or experimental/in vitro models without human transfusion.

**PRISMA justification:** “No transfused population / irrelevant to malaria transfusion risk assessment.”

2. **Methods insufficiently described**

**Description:** Studies where methodology or diagnostic rationale is unclear, making reliable interpretation or extraction of data impossible.

**PRISMA justification:** “Methods described in the article are insufficiently clear for reliable data extraction.”

3. **Full text not accessible**

**Description:** Articles with relevant titles/abstracts but for which full text could not be obtained despite searching institutional databases.

**PRISMA justification:** “Full text not accessible; eligibility assessment and data extraction not possible.”

4. **Duplicate dataset**

**Description:** Articles reporting the exact same data as another retained study, even if authors or publication years differ.

**PRISMA justification:** “Duplicate dataset; data already included in another retained study.”

5. **Data not extractable for meta-analysis**

**Description:** Articles for which quantitative data necessary for meta-analysis are unavailable, despite inclusion of human transfusion recipients. Examples: studies reporting only narrative results, aggregated outcomes without precise numbers, or otherwise insufficient for calculating prevalence/proportions.

**PRISMA justification:** “Quantitative data not extractable for meta-analysis.”

6. **Excluded due to lack of consensus for inclusion**

**Description:** Articles otherwise relevant and adequately described but for which the review team could not reach consensus. Reasons: disagreement on clinical applicability, borderline methodological quality, inconsistencies in interpreting inclusion criteria, or other scientific uncertainties.

**PRISMA justification:** “Excluded due to lack of consensus for inclusion among review team.”

**Consequence:** These studies were potentially relevant but not included in the meta-analysis due to unresolved scientific disagreements

## 7. ****Excluded after regional eligibility filter (contextual ineligibility)****

### **Description:** These studies initially appeared eligible based on title and abstract screening, suggesting relevance to sub-Saharan Africa and transfusion-transmitted malaria. However, during full-text review, it was noted that they were conducted **outside the predefined regional context** of the review.

This may include:

- Studies conducted in populations outside sub-Saharan Africa despite passing initial filters
- Studies involving imported malaria cases or transfusions not relevant to the endemic context of interest
- Cohorts or settings (e.g., military, migrant, or special populations) that do not align with the predefined population criteria

These studies were initially retrieved through regional filters, but full-text examination revealed that they **did not meet the geographical or epidemiological scope** defined in the protocol, so they were excluded.

### **PRISMA Justification:** “Excluded after full-text assessment because the study context fell outside the predefined regional scope.”
